# Domestic Summary

**Published:** 1840-02-15

**Authors:** 


### DOMESTIC SUMMARY.

*The London Dissector.*—The American edition of this work, edited by E. J. Chaisty, M. D., demonstrator of anatomy in the University of Maryland, has recently reached us. It has been noticed in most of the medical journals, some time since, and it will be sufficient for us to express our accordance in the opinion generally entertained, that it appears to be a useful guide to the study of anatomy.

*Professor Brainard's Introductory Lecture.*—A well written, sensible discourse, lately delivered at the Medical Institution of Yale College.

*Louisville Medical Institute.*—This institution appears to have attained a success, quite unexampled in rapidity. The number of matriculants for the current session, the third of its existence, is two hundred and four.

*Death from the inhalation of the vapour of the bi-sulphuret of mercury.*—A patient in the surgical ward of the Louisville Hospital, labouring under a venereal ulcer of the palate, was directed to use mercurial fumigation, which he did, in the manner and to the degree usually practised in the ward, but it proved fatal to him in less than half an hour. A detailed report of the case with the *post mortem* appearances will be given in our next number.—*Western Journal of Medicine and Surgery.*

*Milk sickness*, alias *sick stomach.*—This endemic of the West, to which science has not yet given a name, and even sometimes professes to doubt the existence, continues to attract the attention of the people and country practitioners, in various parts of Ohio, Indiana, Illinois, Kentucky and Tennessee. Sometime since, we received two communications concerning it. One from Miami county, the other, accompanied with specimens of a plant supposed to be the remote cause, from Fayette county, in the State of Ohio. The former by an intelligent, but non-professional gentleman, presents as the poisonous plant, supposed to produce the disease, the *Rhus radicans*; but as this vine grows universally in the West, while the malady in question is limited to particular spots, we cannot concur in the hypothesis. The plant transmitted to us by the latter, is the *Eupatorium ageratoides*, found *every where* in our fertile woods; and therefore not likely to be the cause of a disease perseveringly limited to certain localities. Moreover, judging it by the taste, it is quite inert; and indeed the whole genus to which it belongs, except the *perfoliatum*, appear to be destitute of active qualities, and that species is by no means of a noxious character.

In connexion with this subject we record the following fact. In the month of July last, about twenty of the boarders, in the hotel of Mr. Madeira, Chillicothe, Ohio, were attacked, in one, two, or three hours after breakfast, with nausea and vomiting. In some, the latter was violent, accompanied with spasms of the stomach, and a degree of prostration from which they did not entirely recover for three or four days. Of course, this affection was ascribed to something eaten at the table, but the only article taken by the whole, was *butter*; and that butter, it was ascertained, had been brought from an adjoining county in which the milk sickness prevails. Many facts of this kind have been reported by the people in different parts of the West, but, generally, discredited by the profession. We beg leave to commend the whole subject to our country friends, and shall be happy to give publicity to their observations and experiments.—*Ib.*

*Scarlatina simplex.*—For several years past, different varieties of scarlatina have prevailed somewhere in the West. In one year, one town has been affected, in another, a different place. In degree, it has varied from high and fatal malignancy, to extreme mildness. During the present winter, in Louisville, it has assumed the latter character, presenting itself as a slight febrile affection with moderate tumefaction of the glands and ganglia of the throat and neck, followed in several cases by œdema of the lower extremities. In some families, most of the children have been either simultaneously or successively affected. We shall be happy to receive for publication an account of this epidemic, as it has laterally shown itself in the West.—*Ib.*

[This account of the scarlatina epidemics, illustrates some of the remarks which we have hazarded in our notice of the Journal from which we quote.—Ed.]

